# African Swine Fever Virus Structural Protein p17 Inhibits Cell Proliferation through ER Stress—ROS Mediated Cell Cycle Arrest

**DOI:** 10.3390/v13010021

**Published:** 2020-12-24

**Authors:** Nengwen Xia, Hui Wang, Xueliang Liu, Qi Shao, Da Ao, Yulin Xu, Sen Jiang, Jia Luo, Jiajia Zhang, Nanhua Chen, François Meurens, Wanglong Zheng, Jianzhong Zhu

**Affiliations:** 1 College Veterinary Medicine, Yangzhou University, Yangzhou 225009, China; 13089071533@163.com (N.X.); wanghuixxl@outlook.com (H.W.); lxl1109093817@yeah.net (X.L.); sq970123@163.com (Q.S.); da-ao@outlook.com (D.A.); ylxu15650096726@163.com (Y.X.); jiangsen8888@163.com (S.J.); jialuo20200202@163.com (J.L.); zjj1608291303@163.com (J.Z.); hnchen@yzu.edu.cn (N.C.); 2 Comparative Medicine Research Institute, Yangzhou University, Yangzhou 225009, China; 3 Joint International Research Laboratory of Agriculture and Agri-Product Safety, Yangzhou 225009, China; 4 Jiangsu Co-Innovation Center for Prevention and Control of Important Animal Infectious Diseases and Zoonoses, Yangzhou 225009, China; 5 BIOEPAR, INRAE, Oniris, Nantes 44307, France; francois.meurens@inra.fr

**Keywords:** African swine fever virus, p17 protein, ER stress, ROS, cell cycle

## Abstract

African swine fever virus (ASFV) is a highly pathogenic large DNA virus that causes African swine fever (ASF) in domestic pigs and wild boars. The p17 protein, encoded by the D117L gene, is a major transmembrane protein of the capsid and the inner lipid envelope. The aim of this study was to investigate the effects of p17 on cell proliferation and the underlying mechanisms of action. The effects of p17 on cell proliferation, cell cycle, apoptosis, oxidative stress, and endoplasmic reticulum (ER) stress have been examined in 293T, PK15, and PAM cells, respectively. The results showed that p17 reduced cell proliferation by causing cell cycle arrest at G2/M phase. Further, p17-induced oxidative stress and increased the level of intracellular reactive oxygen species (ROS). Decreasing the level of ROS partially reversed the cell cycle arrest and prevented the decrease of cell proliferation induced by p17 protein. In addition, p17-induced ER stress, and alleviating ER stress decreased the production of ROS and prevented the decrease of cell proliferation induced by p17. Taken together, this study suggests that p17 can inhibit cell proliferation through ER stress and ROS-mediated cell cycle arrest, which might implicate the involvement of p17 in ASF pathogenesis.

## 1. Introduction

African swine fever virus (ASFV) is a highly pathogenic double-stranded DNA virus with a genome size of approximately 180–190 kb. It causes African swine fever (ASF) in some members of Suidae, such as domestic pigs and wild boars, which is currently the most deadly infectious disease affecting the global swine industry [1,2]. ASFV replicates in macrophages or monocytes, and is able to evade immune defenses through multiple strategies [3,4,5,6,7]. ASF pathology is characterized by severe leukopenia and a massive destruction of the lymphoid organs and tissues, including the spleen, lymph nodes, thymus, and tonsils [8,9,10]. The destruction of host immune cells in ASF as a significant immune evasion strategy is likely attributed to cell apoptosis [11], necrosis [12], and autophagy [13]. The ASFV genome contains genes involved in programmed cell death, either in a stimulatory or an inhibitory way [14]. More specifically, ASFV infection of cells was shown to induce apoptosis through mitochondrial caspase 9-caspase 3 pathway, as well as through caspase 12 associated with endoplasmic reticulum (ER) stress [15,16]. ASFV also encodes several apoptosis inhibitor genes, including A224L, A179L, EP153R, and DP71L, which prevent premature cell death and thus support viral replication [17]. In addition, ASFV was also shown to inhibit autophagosome formation through its A179L protein interaction with BH3 homology domain of Beclin1, even though the impact on cell viability was unknown [13,18].

ASFV p17 protein is encoded by the D117L gene and consists of 117 amino acids. P17 is a major structural transmembrane protein localized in the capsid and inner lipid envelope [19]. The capsid p17 was found to be located below the external capsid shell, presumably for stabilizing the viral capsid [20]. In the viral capsid, p17 closely associates with the base domain of major capsid protein p72, with three copies of p17 encircling each p72 capsomer in the inner capsid shell, and firmly anchors p72 capsomers on the inner membrane [21]. The inner membrane p17 is an essential and highly abundant protein required for the assembly of the capsid and icosahedral morphogenesis [22]. Together with other minor capsid proteins p49 and M1249L, and the penton protein (H240R), a complicated network immediately below the outer capsid shell is formed, stabilizing the whole capsid. The complex capsid structure has been recently solved by several groups, clearly demonstrating that the major capsid protein (p72) and four stabilizing minor proteins (H240R, M1249L, p17, p49) are interacting as penta- and tri-symmetrons [19,20,21].

ASFV encodes more than 150 polypeptides, which may have intricate and delicate interactions with the host for the benefit of the virus to evade the host’s defenses. These viral proteins facilitate ASFV replication in infected cells and subsequent viral spreading for further infections. However, currently, there is still a lack of information regarding the roles of the different proteins encoded by ASFV in infected host cells. We analyzed the effects of whole genomic open reading frames (ORFs) of ASFV China 2018/1 on cytotoxicity using 293T and PAM cells, and found that several ORFs, including the D117L gene, could cause cytotoxicity and inhibit cell proliferation. This observation has importance in better understanding ASF pathogenesis. Thus, the main aim of this study was to investigate the impacts and related molecular mechanisms of ASFV p17 encoded by the D117L gene on cell proliferation. In our report, the impacts of p17 on cell proliferation, cell apoptosis, cell cycle arrest, oxidative stress, and ER stress were assessed. The results suggested that ASFV p17 could inhibit cell proliferation through ER stress and ROS-mediated cell cycle arrest, playing a potential role in ASF pathogenesis.

## 2. Materials and Methods

### 2.1. Chemical Reagents and Antibodies

DMEM medium and RPMI 1640 medium were obtained from Hyclone (Hyclone Laboratories, Logan, Utah, USA). Fetal bovine serum (FBS) was obtained from Gibco (Grand Island, NY, USA). A cell counting kit-8 (CCK8) kit was purchased from Dojindo Laboratories (Kumamoto, Kyushu, Japan). An ROS assay kit (S0033), MTT Cell Proliferation and Cytotoxicity Assay Kit (C0009), LDH Cytotoxicity Assay Kit (C0017), and N-acetyl-L-cysteine (NAC, S0077) were obtained from Beyotime Institute of Biotechnology (Shanghai, China), while 4-phenylbutyrate (4-PBA) was from Sigma–Aldrich (St. Louis, MO, USA). PI/RNase Staining Buffer (550825) and Annexin V/propidium iodide (PI) were purchased from Becton Dickinson Company (BD; Franklin Lakes, NJ, USA). The antibodies against β-actin (5057), Cyclin A2 (91500), Cyclin B1 (12231), Cyclin E1 (20808), Geminin (52508), GPR78 (3177), PERK (5683), IRE1α (3294), PDI (3501), Ero1-Lα (3264), and FLAG (14793) were acquired from Cell Signaling Technology (Boston, MA, USA). The antibody against dsRed (ab185921) was acquired from Abcam (Abcam, Cambridge, Cambridgeshire, UK).

### 2.2. D117L/p17 Expression Plasmids

The D117L gene of ASFV China 2018/1 (GenBank submission No. MH766894) was codon optimized for protein expression in mammalian cells, including human 293T cells. It was synthesized and cloned into p3XFLAG-CMV-7.1 vector using *Hind* III and *Kpn* I sites, and pDsRed-Express-C1 vector using *Bgl* II and *EcoR* I sites. The codon optimized DNA sequence of D117L gene is as follow: ATGGACACTGAAACGTCTCCACTGCTTTCTCATAACCTGTCAACCCGCGAGGGAATTAAACAAAGCACCCAAGGCCTTTTAGCCCATACAATCGCCAAATATCCCGGAACAACTGCGATTCTCCTGGGCATTTTGATTTTGCTCATTATTATTCTTATCATCGTTGCCATCGTTTACTATAACCGGACTATTGACTGCAAGTCGAGCATACCTAAACCTCCTCCTAGCTACTATGTACAACAACCTGAGCCTCACCACCATTTCCCGGTATTCTTTAGAAAAAGGAAAAACTCCACCTCCCTGCAGTCCCACATTCCAAGCGACGAACAATTAGCTGAACTTGCGCATTCATAA.

### 2.3. Cell Culture and Transfection

Human embryonic kidneys (293T) and porcine kidney epithelial cells (PK15) were maintained in a DMEM medium supplemented with 10% fetal bovine serum, 1 mM glutamine, and 1% penicillin/streptomycin, and maintained at 37 °C with 5% CO2. Porcine alveolar macrophages (PAMs) were maintained in an RPMI 1640 medium supplemented with 10% fetal bovine serum, 1 mM glutamine, and 1% penicillin/streptomycin, and maintained at 37 °C with 5% CO2. Transfection was performed by using the Lipofectamine 2000 (Invitrogen, Carlsbad, CA, USA) following the manufacturers’ instructions. Cells were seeded in 96-well or six-well plates and cultured in a growth medium one day before. Plasmids and Lipofectamine 2000 were prepared in an Opti-MEM I medium, respectively. After 5 min of incubation, two solutions were mixed gently and incubated for 20 min at room temperature. The plasmid/Lipofectamine 2000 complexes were added to each well containing cells and the medium. The cells were incubated at 37 °C in a CO2 incubator until ready to assay.

### 2.4. Cell Proliferation and LDH Release Analysis

Cell proliferation was analyzed by using CCK8 and MTT kits. The principles of CCK8 and MTT assays are similar. Both of them determine the cell proliferation based on the ability of cells to reduce a tetrazolium dye by the action of NAD(P)H-dependent cellular oxidoreductase, which is an enzyme presenting in the viable cells. However, the tetrazolium dyes used in the CCK8 and MTT assays are different. The tetrazolium dye used in the MTT assay is 3-(4,5-dimethylthiazol-2-yl)-2,5-diphenyltetrazolium bromide, which results in an insoluble formazan product. The CCK8 assay utilizes 3-(4,5-dimethylthiazol-2-yl)-5-(3-carboxymethoxyphenyl)-2-(4-sulfophenyl)-2H-tetrazolium, which results in an aqueous, soluble formazan product. Briefly, the cells were plated in the 96-well culture plates at the density of 5 × 10^4^ cells/each well. Ten µL of the CCK-8 solution was added to the cell culture medium 2 h before the end of treatment. The optical density (OD) of each well was read by the micro-plate absorbance reader at 450 nm. The effects of pP17 on cell proliferation were also determined by using the MTT assay. The cells were plated at a concentration of 5 × 10^4^ cells per well in 96-well culture plates. After cells were treated, 10 µL of 10 mg/mL MTT was added to the cell culture medium. The resulting formazan crystals were dissolved in dimethyl sulfoxide. Absorbance was measured by a microplate spectrophotometer at 490 nm.

Lactate dehydrogenase (LDH) is a cytoplasmic enzyme released upon cell plasma membrane damage and by dying cells. Thus, cytotoxicity was analyzed by monitoring LDH level in the culture medium with a LDH cytotoxicity assay kit according to the manufacturer’s instructions (Beyotime, Shanghai, China). The OD of each well was measured spectrophotometrically at 490 nm with a microplate reader.

### 2.5. Cell Apoptosis and Cell Cycle Distribution Analysis

The level of cell apoptosis was examined using the Annexin V-fluorescein isothiocyanate apoptosis detection kit. Briefly, after treatment, the cells were harvested and washed by the binding buffer, and then re-suspended in the binding buffer. The staining solution of Annexin V-FITC (Fluorescein isothiocyanate isomer I) and PI (propidium iodide) were added one by one. The cells were incubated at room temperature for 15 min without light, and the stained cells were immediately detected using flow cytometry.

Cell cycle distribution was analyzed using PI/RNase Staining Buffer (BD; Franklin Lakes, NJ, USA). Briefly, after cells were subjected to various treatment, the cells were harvested and fixed in 70% ethanol. Fixed cells were stained by the PI/RNase staining buffer for 30 min at room temperature. The stained cells were analyzed for cell cycle using flow cytometry after filtered by using a 200-mesh nylon filter.

### 2.6. Western Blot Analysis

Whole cell proteins were extracted with an RIPA lysis buffer. Then, the concentration of the whole protein was analyzed and adjusted using the BCA protein assay kit (Beyotime Institute of Biotechnology, Shanghai, China). The protein samples were mixed with 1× loading buffer and boiled for 10 min. The protein supernatants were run by SDS-PAGE, and then the proteins in gel were transferred to PVDF membranes. The membranes were incubated with 5% skim milk solution at room temperature for 2 h, probed with the indicated primary antibodies at 4 °C overnight, washed, and then incubated with secondary antibodies for 1 h at RT. The protein signals were detected by ECL detection substrate and imaging system.

### 2.7. Confocal Microscopy

Cells cultured in cell coverslip in a 24-well plate were fixed in 4% paraformaldehyde for 30 min at room temperature, permeabilized by 0.5% Triton X-100, and then blocked with 5% BSA. The treated cells were incubated with primary anti-FLAG Rabbit mAb (#14793, Cell Signaling Technology, Boston, MA, USA) overnight, and then incubated with Goat anti-Rabbit IgG (H + L) Cross-Adsorbed Secondary Antibody, DyLight 488 (#35553, Thermo Fisher, Sunnyvale, CA, USA) for 1 h. Finally, the coverslips were counterstained with DAPI, loaded on slide, sealed by nail polish, and visualized under a confocal laser-scanning microscope (Leica TCS SP8, Leica, Weztlar, Germany). Red fluorescence protein dsRed expression in cells was directly detected by microscope.

### 2.8. Measurement of Cellular Reactive Oxygen Species (ROS) and Ca^2+^

The level of intracellular ROS was detected by using an (ROS) assay kit. After being washed by PBS, cells were re-suspended in PBS with 10 µM of DCFH and incubated for 40 min at 37 °C. Then, the cells were collected and washed twice with PBS. The level of ROS was analyzed by flow cytometry at a wavelength pair of 488/525 nm.

Mitochondrial ROS was measured by using mitoSOX red mitochondrial superoxide indicator (Yeasen Biotech, Shanghai, China). This fluorogenic dye can selectively enter the mitochondria of living cells, in which it is oxidized by superoxide anions. After treatment, cells were harvested and washed twice with PBS. The cells were stained with 2 μM of mitoSOX at 37 °C for 10 min, followed by wash with PBS, and then analyzed by flow cytometry wavelengths 510/580 nm.

The intracellular Ca^2+^ was measured by using Fluo-3 AM (Beyotime Institute of Biotechnology, Shanghai, China). After treatment, cells were harvested and washed twice with PBS. The cells were stained with Fluo-3 AM at 37 °C for 30 min without light, and then used immediately for detection by flow cytometry.

### 2.9. Statistical Analysis

All of the experiments were representative of three similar experiments. The results were analyzed by using SPSS and presented as the mean ± standard deviation (SD). Statistical analysis was performed by using Student’s t-test and ANOVA; *p* < 0.05 was considered statistically significant.

## 3. Results

### 3.1. The Expression of p17 Protein in 293T and PAM Cells

**Figure 1:**
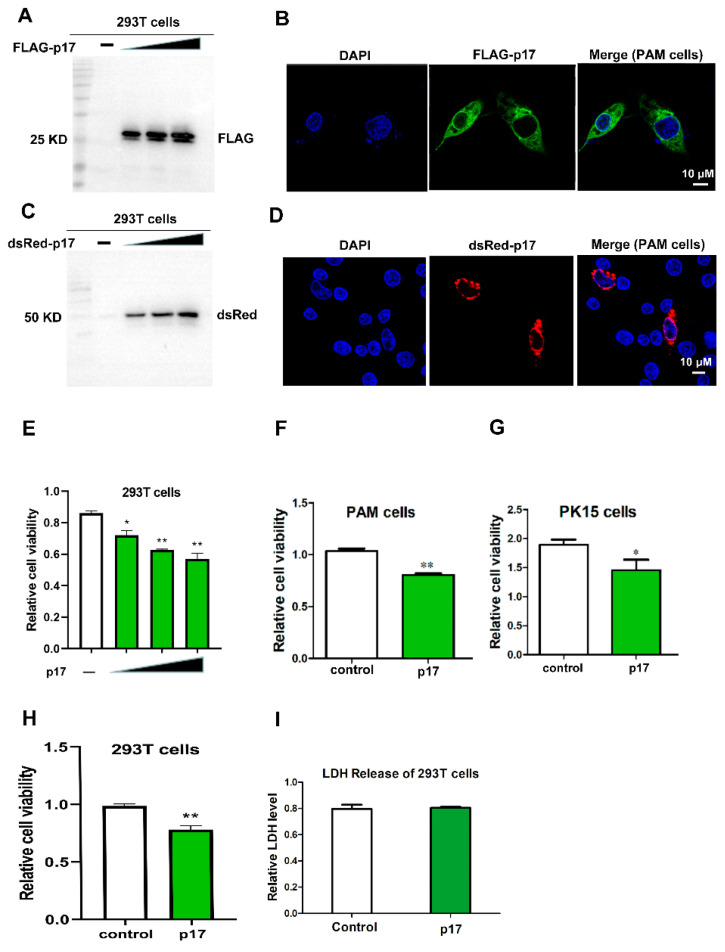
Detecting the expression of ASFV p17 protein in 293T and PAM cells, and effects of p17 on cell proliferation and LDH release. (**A**) Expression of FLAG-p17 protein in 293T cells was detected by Western Blot using anti-FLAG after transfection with FLAG-p17 plasmids for 24 h. The FLAG-p17 plasmids for transfection were 0.25, 0.5, and 1 μg/mL, respectively. (**B**) Expression of FLAG-p17 protein in PAM cells by IF. (**C**) Expression of dsRed-p17 protein in 293T cells was detected by western blot using anti-dsRed after transfection with dsRed-p17 plasmids for 24 h. The dsRed-p17 plasmids for transfection were 0.25, 0.5, and 1 μg/mL, respectively. (**D**) Expression of dsRed-p17 protein in PAM cells was detected by IF. (**E**) Effects of p17 on cell proliferation in 293T cells were analyzed with a CCK-8 kit after transfection with FLAG-p17 plasmids for 24 h. The FLAG-p17 plasmids for transfection were 0.25, 0.5, and 1 μg/mL, respectively. (**F**,**G**) Effects of p17 on cell proliferation in PAM and PK15 cells were analyzed with a CCK-8 kit. The p17 groups were transfected with FLAG-p17 (1 μg/mL) for 24 h. (**H**) Effect of p17 on cell proliferation in 293T was detected with an MTT assay. The FLAG-p17 plasmid for transfection was 1 μg/mL. (**I**) Effect of p17 on LDH release in 293T cells. The p17 groups wasere transfected with FLAG-p17 (1 μg/mL) for 24 h. Control groups were transfected with empty plasmids. Values represent the mean ± S.D; * *p* < 0.05, ** *p* < 0.01 versus the control group.

The plasmids of FLAG and dsRed tagged ASFV D117L genes were transfected into 293T and PAM cells, respectively, using lipofectamine 2000. Expression of p17 protein was examined by western blot (WB) and immunofluorescence (IF) assay. As shown in Figure 1A,C, the sizes of FLAG-p17 and dsRed-p17 were around 25 kD and 48 kD, respectively. Both of them were larger than the expected sizes, which may be attributed to the posttranslational modifications. The results from IF showed that ASFV p17 was mainly localized in the cytoplasm of PAM cells (Figure 1B,D).

### 3.2. Effects of p17 Expression on Cell Proliferation and LDH Release

The effect of p17 protein on cell proliferation was evaluated using CCK8 and MTT assays in transfected 293T, PAM, and PK15 cells. The results from the CCK8 and MTT assays showed that, compared with the control groups, cell proliferation significantly decreased (*p* < 0.05 and *p* < 0.01) in the cells expressing p17 (Figure 1E–H). Cell death was measured by the quantification of LDH released into the supernatant. However, the results showed that the level of LDH in the cell culture supernatant did not exhibit a significant difference between the p17 expressing cells and control cells (Figure 1I). These data suggested that ASFV p17 could suppress cell proliferation while not significantly affecting LDH release.

### 3.3. Effects of p17 on Cell Apoptosis and Cell Cycle Progression

**Figure 2:**
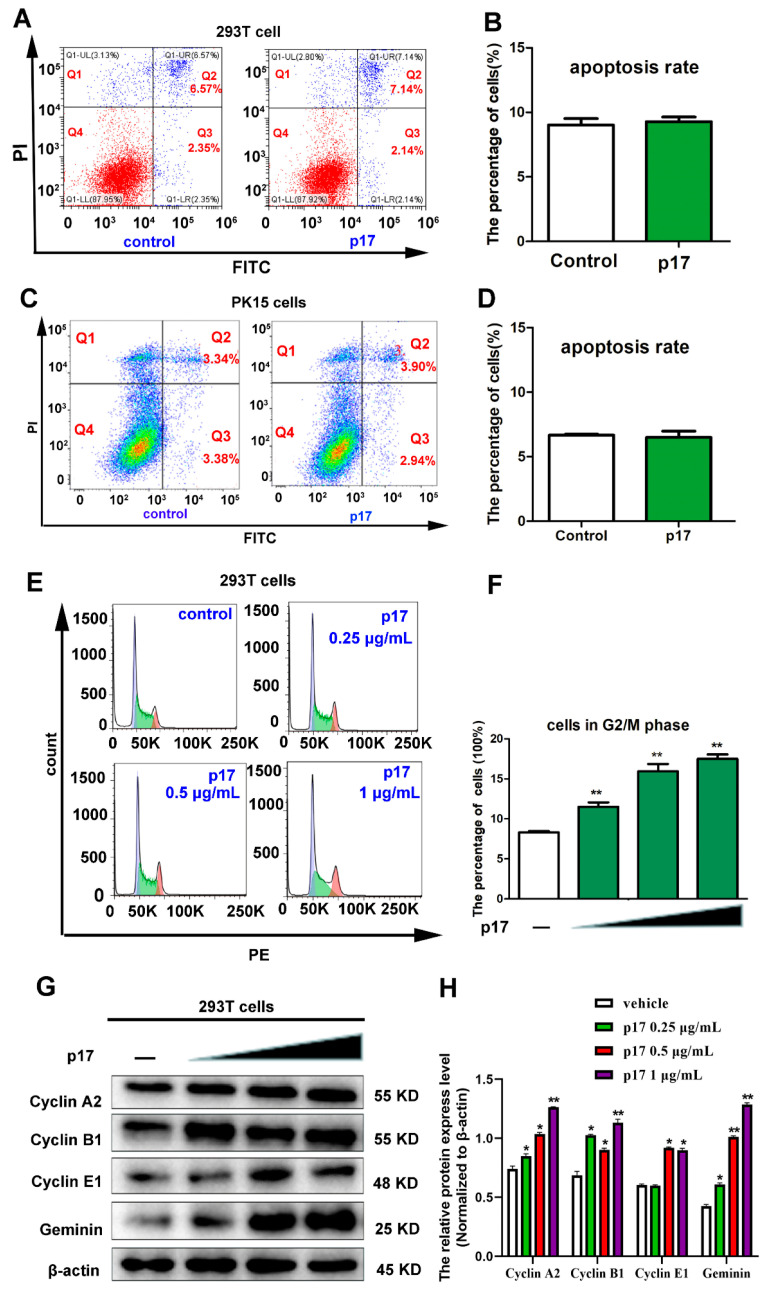
Effects of p17 on cell apoptosis and cell cycle progression. (**A**–**D**) The effects of p17 on cell apoptosis in 293T and PK15 cells. The effects of p17 on cell apoptosis were analyzed using Annexin V-FITC/PI staining followed by flow cytometry. Control groups were transfected with empty plasmid for 24 h. P17 groups were transfected with FLAG-p17 plasmid (1 μg/mL) for 24 h. (**E**,**F**) The effect of p17 on cell cycle distribution in 293T cells. The cell cycle distribution was analyzed using PI/RNase staining followed by flow cytometry. Control groups were transfected with empty plasmid for 24 h. The FLAG-p17 plasmids for transfection were 0.25, 0.5, and 1 μg/mL, respectively, for 24 h. (**G**) Effect of p17 on the expressions of cell cycle regulatory proteins, including Cyclin A2, Cyclin B1, Cyclin E1, and Geminin. The FLAG-p17 plasmids for transfection were 0.25, 0.5, and 1 μg/mL for 24 h, respectively. (**H**) The intensity of the western blot bands was quantified by densitometric analysis. Values represent the mean ± S.D; * *p* < 0.05, ** *p* < 0.01 versus the control groups.

To examine the cellular processes of p17-reduced cell proliferation, the effects of p17 on cell apoptosis in 293T and PK15 cells were analyzed using annexin V staining, followed by flow cytometry. As shown in Figure 2A–D, compared with the control groups, the ratios of cell apoptosis in p17 transfected cells was not altered significantly. To further explore the molecular mechanisms of p17-reduced cell proliferation, the impacts of p17 on cell cycle distribution and expressions of cell cycle regulatory proteins were analyzed. As shown in Figure 2E,F, p17 caused a notable accumulation of 293T cells in G2 phase cells in a dose-dependent manner. The results from western blot showed that, compared with the control group, the protein levels of cell cycle regulatory proteins, including Cyclin A2, Cyclin B1, Cyclin E1, and Geminin, were all increased in a dose-dependent way in 293T cells transfected with p17 gene (Figure 2G,H). These data suggest that ASFV p17 has no effects on cell apoptosis, but causes a cell cycle arrest at the G2/M phase.

### 3.4. ASFV p17-Induced Cell Cycle Arrest Through the Production of Reactive Oxygen Species (ROS)

**Figure 3:**
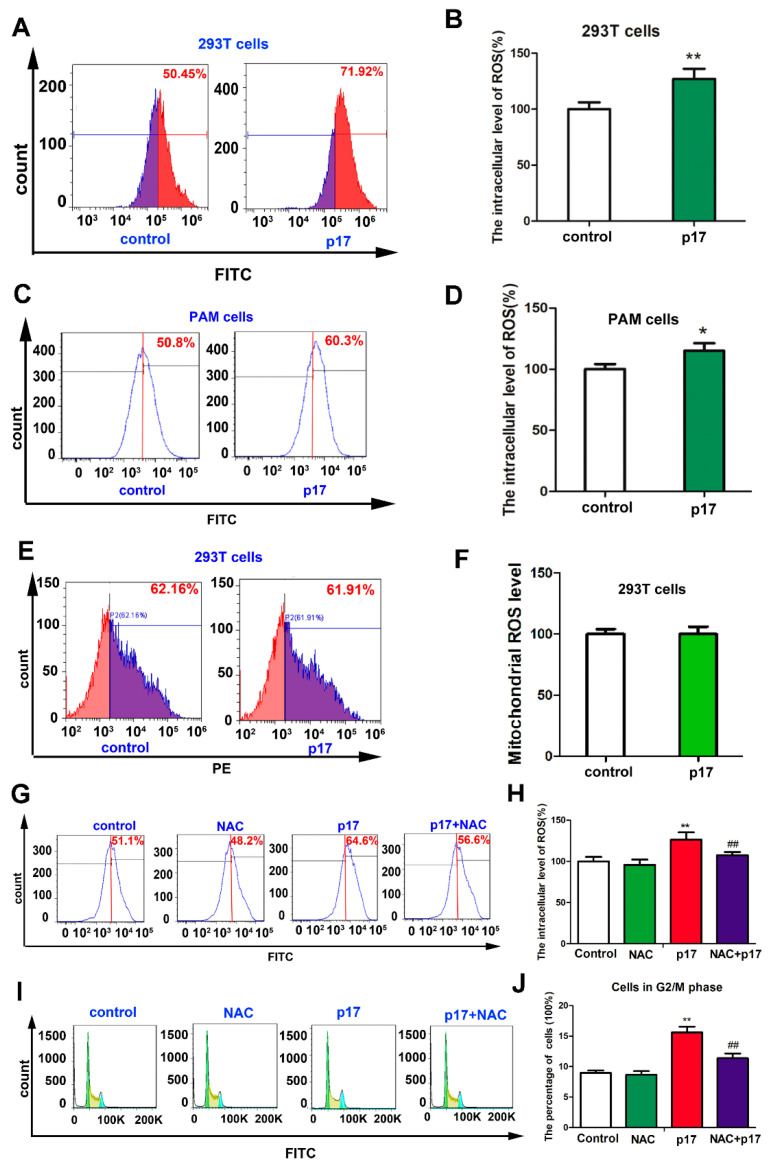
P17 could induce the production of ROS, and the intracellular ROS was involved in the process of p17-induced cell cycle arrest. (**A**–**D**) p17 induced the overproduction of intracellular ROS in 293T and PAM cells. After cells were transfected with FLAG-p17 (1 μg/mL) plasmids for 24 h, the cells were collected and the intracellular ROS level was analyzed with 2′,7′-dichlorofluorescin diacetate staining followed by flow cytometry. (**E**,**F**) The effect of p17 on the mitochondrial level of ROS in 293T cells. After cells were transfected with FLAG-p17 (1 μg/mL) plasmids for 24 h, the mitochondrial level of ROS was analyzed using a mitoSOX red mitochondrial superoxide indicator followed by flow cytometry. (**G**,**H**) NAC could alleviate the overproduction of ROS induced by p17. 293T cells were pretreated with NAC (2.5 mM) for 30 min, and then transfected with p17 gene plasmid in the presence of NAC for another 24 h. (**I**,**J**) NAC (2.5 mM) could partly reverse the cell cycle arrest induced by p17. Control groups were transfected with empty plasmids; * *p* < 0.05, ** *p* < 0.01 versus control groups; ^##^ *p* < 0.01 versus p17 groups.

In order to determine the underlying molecular events of p17-induced cell cycle arrest, the intracellular and mitochondrial levels of ROS were analyzed using flow cytometry. As shown in Figure 3A–D, compared with control groups, the intracellular levels of ROS in both 293T and PAM cells transfected with p17 gene were significantly increased (*p* < 0.01 and *p* < 0.05). However, compared to control group, the level of ROS in mitochondria did not exhibit significant change upon p17 expression in 293T cells (Figure 3E,F). Then, we examined whether intracellular ROS was involved in the process of p17-induced cell cycle arrest. As shown in the Figure 3G,H, the antioxidant N-acetyl-L-cysteine (NAC) was used, which could alleviate the overproduction of ROS induced by p17 expression. Treatment with NAC could partially reverse the cell cycle arrest induced by p17 (Figure 3I,J). These data suggest that the overproduction of ROS was involved in the process of p17-induced cell cycle arrest.

### 3.5. ASFV p17 Was Localized in the Endoplasmic Reticulum (ER) and Induced ER Stress

**Figure 4:**
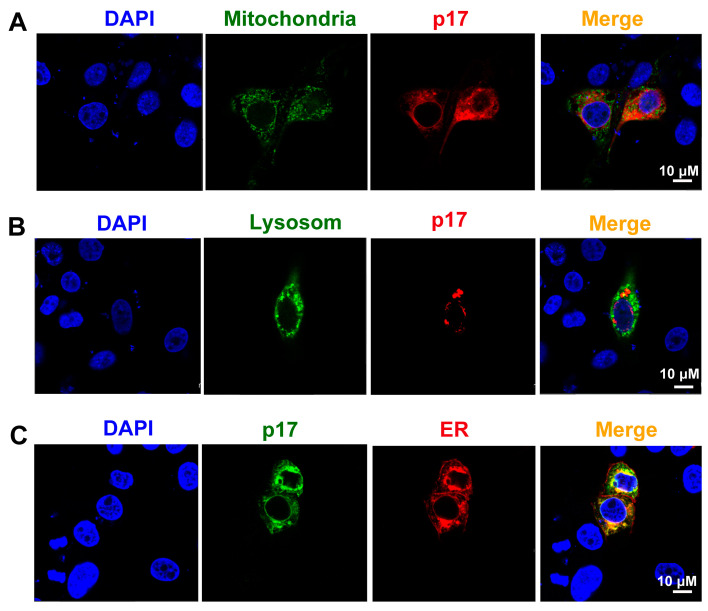
The subcellular localization of p17 protein in PAM cells was determined by confocal microscopy; p17 protein was co-localized with the ER marker, but not with mitochondrion and lysosome markers. (**A**) Plasmids of dsRed-p17 and Mito-EGFP (mitochondrial targeting sequence of cytochrome c oxidase subunit VII fused to EGFP) were co-transfected into PAM cells. (**B**) Plasmids of dsRed-p17 and GFP-LAMP1 (lysosomal-associated membrane protein1) were co-transfected into PAM cells. (**C**) Plasmids of FLAG-p17 and RFP-KDEL (an ER retention protein) were co-transfected into PAM cells.

To explore the mechanisms of p17-induced the production of ROS, the subcellular localization of p17 in PAM cells was determined by confocal microscopy. The results showed that p17 protein was co-localized with the KDEL ER retention RFP marker, but not with the mitochondria-targeting EGFP marker and lysosome LAMP1-GFP marker (Figure 4A–C).

**Figure 5:**
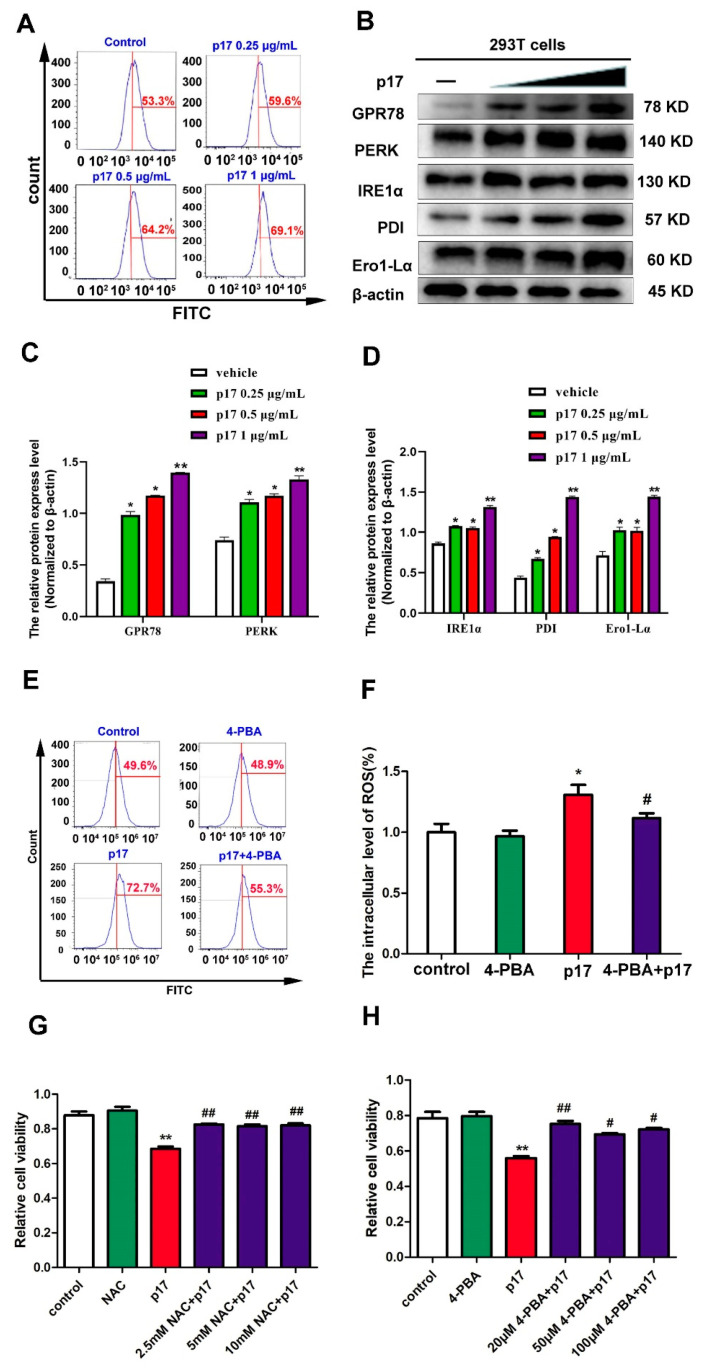
P17 could induce ER stress, and ER stress was responsible for the p17-inhibited cell proliferation. (**A**) The effect of p17 on the level of intracellular Ca^2+^ was analyzed using Fluo-3 AM staining followed by flow cytometry. P17 groups were transfected with FLAG-p17 plasmids (0.25, 0.5, and 1 μg/mL) for 24 h. (**B**) The effect of p17 on the expressions of GPR78, PERK, IRE1α, PDI, and Ero1-Lα. P17 groups were transfected with FLAG-p17 plasmids (0.25, 0.5, and 1 μg/mL) for 24 h. (**C**,**D**) The intensity of the western blot bands was quantified by densitometric analysis. (**E**,**F**) 4-PBA pre-treatment could decrease the production of ROS by p17. 293T cells were pretreated with 4-PBA (20 μM) for 30 min, and then transfected with p17 gene in the presence of 4-PBA for another 24 h. (**G**) The role of ROS in the decrease of cell proliferation induced by p17 was analyzed by using the CCK8 assay. Using NAC (2.5 mM) to alleviate the production of ROS could reverse the decrease of cell proliferation induced by p17 in 293T cells. 293T cells were pretreated with NAC for 30 min, and then transfected with P17 gene in the presence of 4-PBA for another 24 h. (**H**) The role of ER stress in the decrease of cell proliferation induced by p17 was analyzed by using the CCK8 assay. Using 4-PBA (20 μM) to alleviate ER stress could also prevent the cell proliferation decrease induced by p17. Control groups were transfecting with empty plasmids. Values represent the mean ± SD; * *p* < 0.05, ** *p* < 0.01 versus control groups; ^#^ *p* < 0.05, ^##^ *p* < 0.01 versus p17 groups.

Since the protein of p17 was localized within the ER and able to induce the production of ROS, the effect of p17 on ER stress was evaluated. Several molecular indicators of ER stress, including the level of intracellular Ca^2+^ and the expressions of GPR78, PERK, IRE1α, PDI, and Ero1-Lα, were analyzed using flow cytometry and western blotting, respectively. The data from flow cytometry analysis demonstrated that, compared with the control group, the level of intracellular Ca^2+^ was increased in a dose-dependent manner in the cells transfected with p17 gene (Figure 5A). Meanwhile, the protein expressions of GPR78, PERK, IRE1α, PDI, and Ero1-Lα were increased in cells transfected with p17 gene in a dose-dependent manner as well (Figure 5B–D). These results suggest that p17, localized within the ER, could induce ER stress.

### 3.6. ASFV p17 Inhibited Cell Proliferation through ER Stress and ROS-Mediated Cell Cycle Arrest

In order to explore the role of ER stress in p17-induced production of ROS and cell proliferation, the ER stress inhibitor 4-phenyl butyric acid (4-PBA) was used to alleviate the ER stress signal. The data from flow cytometry analysis showed that 4-PBA pre-treatment decreased the production of ROS induced by p17 (Figure 5E,F). Moreover, the data from the CCK8 assay showed that NAC used to alleviate the production of ROS reversed the decrease of cell proliferation induced by p17 (Figure 5G); 4-PBA, used to alleviate ER stress, also prevented the cell proliferation decrease induced by p17 (Figure 5H). Taken together, these data suggest that p17 induces the production of ROS through ER stress, which further inhibits cell proliferation through cell cycle arrest.

## 4. Discussion

Viruses are obligate intracellular microorganisms that constantly evolve strategies to subvert their hostile cellular environment. As the survival of viruses depends on the ability to replicate in living cells, it is not surprising that they are able to arrest or promote cell cycle progression, depending on the purpose, to their advantage [23]. Previous studies have revealed that ASFV could elicit the Ataxia Telangiectasia mutated and Rad3-related protein-Checkpoint kinase 1 (ATR-Chk1) pathway, which is required for successful viral infection of host cells [24]. Activation of the ATR-Chk1 pathway has been associated with cellular S phase and G2/M checkpoint arrest, which may benefit the virus to gain extra time to recruit host cellular factors for its replication [24]. Moreover, a previous study has indicated that ASFV could recruit specific host translation factors, including 4E-BP1, eIF4E, and eIF4G, into the viral factories to gain extra advantage for its replication [25]. Together, these reports strongly support the hypothesis that ASFV could manipulate cell cycle progression in infected cells, which may help ASFV to recruit specific host factors and to gain extra time for its replication. However, direct evidence for the effects of ASFV on cell cycle progression is lacking. In the current study, the results show that the ASFV structural protein p17 could decrease cell proliferation, other than cause cell death or apoptosis (Figure 1 and Figure 2A–D). Instead, our study also suggests that p17 can cause cell cycle arrest and affect the expressions of cell cycle regulatory proteins, including Cyclin A2, Cyclin B1, CyclinE1, and Geminin (Figure 2E–H). Studies have indicated that Cyclin A2, Cyclin B1, and Cyclin E1 peak at the G2 phase, late G2/M phase, and late G1/early S phase, respectively [26]. Cyclin A2 drives the progression through the S and G2/M phases of the cell cycle. Cyclin B1 is expressed in both mitotic and meiotic cells, and it plays an indispensable role in the G2/M phase. It has been reported that Cyclin E1 is a functional redundancy regulator [27]. Geminin is degraded and downregulated in the G1 phase and late S phase. Geminin is required to ensure once-per-cell-cycle genome replication, and is implicated in DNA replication control [28,29]. To our knowledge, this is the first time that ASFV p17 is reported to affect cell proliferation by regulating cell cycle progression, which may play an important role in viral pathogenesis.

Accumulated evidence has shown that overproduction of intracellular ROS could affect the mitotic process and cause cell cycle arrest [30,31]. Therefore, we assumed that elevating intracellular levels of ROS production could be the mechanism used by p17 to induce cell cycle arrest. The major producer of ROS is NADPH oxidase complexes, which are distributed in cell plasma membranes, mitochondria, peroxisomes, and the ER [32,33]. Mitochondria and the ER are considered as the major organelles responsible for the production of ROS in the cells [34]. The results from our study suggest that p17 can significantly increase the intracellular level of ROS production but not the ROS level in mitochondria (Figure 3A–F). Thus, the mitochondria would not be the source of ASFV p17-induced ROS.

Recently, emerging evidences indicated that ER stress is an alternative important pathway for the production of ROS [35]. Many viruses use the ER as a site of replication and/or envelope development, and these events can lead to the activation of ER stress [36]. A previous study has demonstrated that ASFV is assembled on the cytoplasmic face of the ER and ultimately enveloped by ER membrane cisternae [37]. Our data indicated that p17 protein is localized within the ER and is able to induce ER stress (Figure 4 and Figure 5A–D). Several studies have revealed that the proteins PDI and Ero1-Lα have a strong association with protein loading in the ER and can trigger ROS generation. In the pathway of oxidative proteins, the electron is transferred from PDI to molecular oxygen and Ero1-Lα using a flavin adenine dinucleotide (FAD)-dependent reaction [38]. ASFV p17 caused the increase of expressions of PDI and Ero1-Lα protein (Figure 5B), which may be responsible for subsequent ROS production. ER stress inhibitor 4-PBA to alleviate ER stress signals prevented the production of ROS induced by p17 (Figure 5E,F). This observation suggests that p17 induces the production of ROS through the ER stress pathway. Additionally, the ER stress inhibitor 4-PBA to alleviate ER stress signals reversed the decrease of cell proliferation induced by p17 (Figure 5H). In the meantime, NAC to alleviate the production of ROS also prevented the decrease of cell proliferation and cell cycle arrest induced by p17 (Figure 3G–J and Figure 5G). These data collectively suggest that p17 inhibit cell proliferation through ER stress and ROS-mediated cell cycle arrest.

## 5. Conclusions

In summary, this study revealed that ASFV p17 protein inhibits cell proliferation by inducing cell cycle arrest via ER stress—ROS pathway. As one of the major structural viral proteins, the action of p17 may play an important role in the pathogenesis of ASF, and thus the understanding of p17’s mechanisms of action might provide deep insights into ASF pathogenesis.

## Data Availability

The data presented in this study are available in insert article.
